# Differences between memory encoding and retrieval failure in mild cognitive impairment: results from quantitative electroencephalography and magnetic resonance volumetry

**DOI:** 10.1186/s13195-020-00739-7

**Published:** 2021-01-04

**Authors:** Su-Hyun Han, Jung-Min Pyun, Soeun Yeo, Dong Won Kang, Ho Tae Jeong, Seung Wan Kang, SangYun Kim, Young Chul Youn

**Affiliations:** 1 Department of Neurology, Chung-Ang University College of Medicine, 102, Heukseok-ro, Dongjak-gu, Seoul, 06973 Republic of Korea; 2 Department of Neurology, Seoul National University Bundang Hospital, Seoul National University College of Medicine, Seongnam, Republic of Korea; 3 iMediSync Inc., Seoul, Republic of Korea; 4 Data Center for Korean EEG, College of Nursing, Seoul National University, 103 Daehak-ro, Jongno-gu, Seoul, 03080 Republic of Korea; 5 Department of Medical Informatics, Chung-Ang University College of Medicine, Seoul, Republic of Korea

**Keywords:** Mild cognitive impairment, Encoding, Retrieval, EEG, MRI, qEEG, Voxel-based morphometry

## Abstract

**Background:**

The memory impairments in mild cognitive impairment (MCI) can be classified into encoding (EF) and retrieval (RF) failure, which can be affected by underlying pathomechanism. We explored the differences structurally and functionally.

**Methods:**

We compared quantitative electroencephalography (qEEG) power spectra and connectivity between 87 MCI patients with EF and 78 MCI with RF using iSyncBrain® (iMediSync Inc., Republic of Korea) (https://isyncbrain.com/). Voxel-based morphometric analysis of the gray matter (GM) in the MCI groups and 71 cognitive normal controls was also done using the Computational Anatomy Toolbox 12 (http://www.neuro.uni-jena.de/cat/).

**Results:**

qEEG showed higher frontal theta and lower beta2 band power, and higher theta connectivity in the EF. There was no statistically significant difference in GM volume between the EF and RF. However, when compared to normal control, GM volume reductions due to EF in the left thalamus and bilateral hippocampi and reductions due to RF in the left thalamus, right superior frontal lobe, right superior temporal lobe, and right middle cingulum were observed (*p* < 0.05, family-wise error correction).

**Conclusions:**

MCI differs functionally and structurally according to their specific memory impairments. The EF findings are structurally and functionally more consistent with the prodromal Alzheimer’s disease stage than the RF findings. Since this study is a cross-sectional study, prospective follow-up studies are needed to investigate whether different types of memory impairments can predict the underlying pathology of amnestic MCI. Additionally, insufficient sample size may lead to ambiguous statistical findings in direct comparisons, and a larger patient cohort could more robustly identify differences in GM volume reductions between the EF and the RF group.

## Background

Mild cognitive impairment (MCI) is considered an intermediate stage in the trajectory from normal cognition to dementia [1]. MCI is a heterogeneous disorder with different prognosis from progression to Alzheimer’s disease (AD) or non-AD dementias to the maintenance or even improvement of cognitive decline [2]. The early recognition of disease progression to AD in patients with MCI is an important topic of interest for clinicians in terms of early intervention and patient education [3, 4]. Because the preclinical or prodromal stage of AD has become a major focus in research regarding disease-modifying therapy, identifying individuals at the risk of developing AD would be needed for researchers [3, 4]. The evidence of Alzheimer pathology in cerebrospinal fluid (CSF) or positron emission tomography (PET) and neurodegeneration in multimodal neuroimaging may provide information about disease progression [5]. However, these biomarkers are not easily accessible due to high cost, invasiveness, and restricted availability. It is impossible to perform these studies on all patients with MCI, and simpler methods may be more valuable in practice.

MCI is subdivided into non-amnestic and amnestic types and single and multiple domains [6]. Many studies have suggested that the subtypes have different etiologies and future outcomes and risk of progress to AD may be influenced by the subtype of MCI [7]. There are different rates of progression among subtypes of MCI [2], and approximately 80% of patients with amnestic MCI convert to AD dementia within 6 years [8]. This subtype classification of MCI may have utility as an easily accessible tool, and additional classifications based on AD pathology to amnestic MCI may be more successful. Memory impairment patterns can be divided into two subtypes that show either encoding (EF) or retrieval failure (RF) [9]. Since it has been suggested that EF originates from hippocampal dysfunctions such as those observed in AD and that RF is rather caused by frontal or subcortical dysfunctions [10, 11], we hypothesized that patients with EF among amnestic MCI are more likely to convert to AD than patients with RF. However, the subtyping of amnestic MCI into EF and RF has drawn little attention so far. Although several studies have investigated clinical characteristics or prognostic values of EF and RF in amnestic MCI [12–14], there is no comprehensive approach to understanding the clinical significance.

Currently, electroencephalography (EEG) and magnetic resonance imaging (MRI) volumetry are extensively studied as a predictive factor of clinical progression to AD [5, 15]. EEG power density, functional coupling, spectral coherence, synchronization, and connectivity provided their clinical efficacy in disease progression to AD [16]. EEG coherence has been studied as a measure of brain connectivity [17], and the imaginary part of coherency (iCoh) has been introduced as a robust method to avoid volume conduction artifacts [18]. Additionally, atrophic patterns including the volume of the hippocampus may be a good biomarker of AD [19–22]. Therefore, we aimed to explore functional and structural differences between EF and RF by these potential neuropathologic biomarkers of AD, and to identify that EF patients may exhibit a more similar pattern to AD compared to RF patients. We used the power spectral, iCoh in EEG, and MRI volumetry analyses as neuropathologic biomarkers of AD. Our findings may suggest the clinical implication of subdivision of amnestic MCI into EF and RF, and may be the basis for future prospective research which investigates biomarker of developing AD.

## Subjects and methods

### Subjects

This retrospective study used the qEEG and three-dimensional T1-weighted MRI (3D T1 MRI) data of patients who visited the Chung-Ang University Hospital Department of Neurology from January 2012 to May 2019 and were diagnosed with single-domain amnestic MCI. This study was approved by the institutional review board of our center (IRB number 1802-004-16143). Written informed consent was obtained from all participants.

**Fig. 1:**
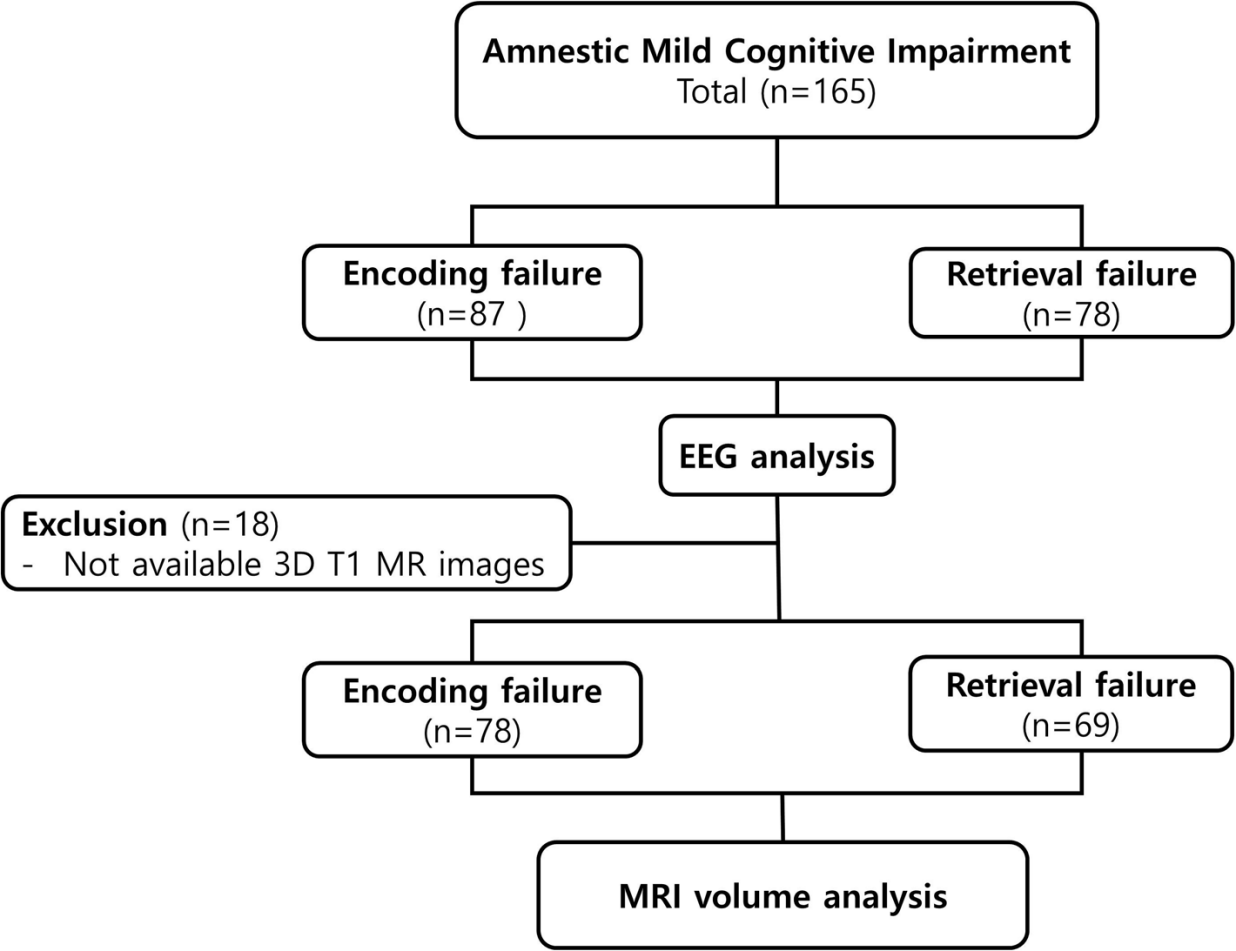
Enrollment of subjects with amnestic mild cognitive impairment

Participants were aged 55 years or older, underwent 3D T1 MRI and qEEG within 2 weeks, and met the single-domain amnestic MCI criteria. The criteria were as follows: (1) presence of memory complaints, (2) intact performance of activities of daily living, (3) objective verbal memory impairments on the Seoul Neuropsychological Screening Battery (at least 1.0 SD below age- and education-adjusted norms), (4) Clinical Dementia Rating of 0.5 (1), and (5) not demented according to the Diagnostic and Statistical Manual of Mental Disorders (DSM)-IV criteria. Subjects were divided into an EF MCI and an RF MCI group. EF was defined as both delayed recall and recognition scores on a verbal learning test below 1.0 SD; RF was defined as only a delayed recall score below 1.0 SD. Resting-state EEG data were obtained from all 165 patients with amnestic MCI comprising 87 with EF and 78 with RF. 3D T1 MRI data were available for brain volume analysis for 147 of all subjects with amnestic MCI (for 78 with EF and 69 with RF) because of problems in the preprocessing of images (Fig. 1).

The 3D T1 MRI imaging data of 71 age-matched cognitive normal control (NC) subjects were selected from the repository. The inclusion criteria for NCs were as follows: (1) from a community-based population; (2) no abnormalities based on a health screening questionnaire [11]; (3) absence of memory complaints; (4) a Korean Dementia Screening Questionnaire score ≤ 6 [12]; (5) a Mini-Mental State Examination (MMSE) score > 26; (6) intact activities of daily living (K-IADL ≤ 0.42); (7) no history of thyroid dysfunction, vitamin B12 deficiency, or folate deficiency; and (8) at least 6 years of education.

No participant presented any structural abnormalities on MRI, such as territorial infarctions, intracranial hemorrhage, brain tumors, or hydrocephalus; lacunar infarcts or mild to moderate subcortical or periventricular white matter hyperintensities did not lead to exclusion. Patients with major psychiatric disease, such as schizophrenia, major mood disorder, and chronic alcoholics, were also excluded.

### qEEG analysis

Resting-state EEG was conducted using the standard 10–20 system (21 electrodes) and a digital electroencephalograph (Comet AS40 amplifier EEG GRASS; Telefactor, USA) (Jaspers, 1958), and all electrodes were referred to linked ear references. Electrode skin impedance was always below 5 kΩ. The EEG signal was analog-filtered with a band pass of 0.5–70 Hz and digitized and stored on magnetic disks for further analysis. EEG sampling was conducted with eyes open for 30 s and with eyes closed for 30 s, 10 times, at a rate of 200 Hz. Of these, about 3 min of eyes-closed data was used. One epoch is 4 s long, and an average of 45 epochs were analyzed. The measured eyes-open and eyes-closed data were converted according to the linked ear reference and stored in text format without filtering. While resting-state EEG data were recorded, patients were lying down in a resting position in a sound-attenuated room. EEG noise preprocessing and group analyses were conducted using iSyncBrain® (iMediSync Inc., Republic of Korea) (https://isyncbrain.com/), a cloud-based, artificial intelligence EEG analysis platform. The eyes-closed EEG segments were uploaded to iSyncBrain®. Prior to data analysis, artifacts in the raw data were removed by visual inspection and an adaptive mixture independent component analysis (amICA) [13]. qEEG features were obtained at the sensor and at the source level. At the sensor level, relative power at eight frequency bands (delta [1–4 Hz], theta [4–8 Hz], alpha1 [8–10 Hz], alpha2 [10–12 Hz], beta1 [12–15 Hz], beta2 [15–20 Hz], beta3 [20–30 Hz], and gamma [30–45 Hz]) was calculated using a power spectrum analysis. In the source-level analysis, the current distribution across the brain was assessed using the standardized low-resolution brain electromagnetic tomography technique [23], to compare relative power values in 8 regions of interests (ROIs) [24] and connectivity (the imaginary part of coherency) [18] between ROIs. Eight ROIs included bilateral temporal lobe, frontal lobe, parietal lobe, and occipital lobe. EEG coherence has been studied as a measure of brain connectivity [17], and the imaginary part of coherency (iCoh) has been introduced to avoid volume conduction artifacts [18]. The iCoh is defined as follows [18]:

1 $$ iCoh= im\left( Coh(f)\right)= im\left(\frac{S_{xy}(f)}{{\left({S}_{xx}(f)\times {S}_{yy}(f)\right)}^{\frac{1}{2}}}\right) $$

where *S*_*xy*_(*f*) is the cross-power spectral density and *S*_*xx*_(*f*) and *S*_*yy*_(*f*) are the autopower spectral densities for each channel *X* and *Y*, respectively. We calculated the connectivity of each of the regional pairwise of 8 ROIs with remaining all other 7 ROIs. We have estimated the functional connectivity at eight frequency bands (delta [1–4 Hz], theta [4–8 Hz], alpha1 [8–10 Hz], alpha2 [10–12 Hz], beta1 [12–15 Hz], beta2 [15–20 Hz], beta3 [20–30 Hz], and gamma [30–45 Hz]).

### MRI volumetry

To determine gray matter (GM) volume changes underlying EF and RF in amnestic MCI, we conducted voxel-based morphometry (VBM) on MRI scans acquired on 3-T scanners manufactured by Philips (Achieva, Amsterdam, the Netherlands). The data were analyzed using the Computational Anatomy Toolbox (CAT12) running on Statistical Parametric Mapping software (SPM12). CAT12 is a VBM toolbox designed by the Structural Brain Mapping Group at the University of Jena (Jena, Germany). First, the DICOM files were converted into nifti format, using MRICRON software (http://people.cas.sc.edu/rorden/mricron/index.html). VBM preprocessing was performed using the default settings of the CAT12 toolbox and the “East Asian Brains” ICBM template. Imaging files were normalized using an affine model, followed by non-linear registration, corrected for bias field inhomogeneities, and then segmented into GM, white matter (WM), and cerebrospinal fluid (CSF) components. The segmented scans were normalized into standard Montreal Neurological Institute space using the Diffeomorphic Anatomic Registration Through Exponentiated Lie (DARTEL) algebra algorithm. The modulation process on the normalized, segmented images consisted of a non-linear deformation, which corrects individual differences in brain size. We reviewed morphological abnormalities and applied smoothing processes to all segmented, modulated, and normalized GM images using an 8-mm full-width-half-maximum Gaussian filter.

### Statistical analysis

To compare demographic and cognitive assessment results between groups, Student’s *t* tests for continuous variables were performed with IBM SPSS version 25 (IBM, Armonk, NY, USA). Statistical significance was set at *p* < 0.05. Student’s *t* test was performed for the frequency band power of each channel and 8 ROIs, and iCoh between 8 ROIs to compare the EF and RF groups. All statistical processes for qEEG features were implanted in iSyncBrain® (iMediSync Inc., Seoul, Republic of Korea).

To demonstrate GM volume changes underlying EF and RF in amnestic MCI, we conducted a comparison with processed MR images of cognitively normal subjects using voxel-wise, two-sample *t* tests of the VBM on SPM package. Age and total intracranial volume (TIV), that is, the sum of the GM, WM, and CSF volumes, were classified as nuisance covariates in the GM volume comparisons between the groups. We used a VBM analysis to demonstrate significant atrophic GM areas in the two types of patients with amnestic MCI. To detect GM volume differences between patients with EF and those with RF, voxel-wise, two-sample *t* tests of the VBM on SPM package were also conducted on the processed images. Age and TIV were again classified as nuisance covariates in the GM volume comparisons between groups. Absolute threshold masking was used at a threshold of 0.1. Results were corrected for family-wise errors (FWE) to avoid multiple-comparison problems of voxel-wise analysis. At cluster level, significant results were displayed at a voxel-wise threshold of *p* < 0.05 with a minimum cluster size (*k*) of 50 voxels. At a peak level, *p* < 0.05 was set as a threshold for significance.

## Results

### Study subjects

The mean age of all subjects with amnestic MCI was 73.5 years, and their mean MMSE score was 22.4. There was no significant difference between the EF and RF groups in baseline demographics or clinical status (*p* > 0.05, Table 1) except for gender (*p* = 0.010). The mean age of all patients with EF was 73.8 ± 8.3 years, that of the patients with RF was 73.4 ± 6.2 years, and that of the NCs (*n* = 71) was 70.4 ± 3.9 years. There was also no significant difference between the EF and RF groups in the score of Clinical Dementia Rating (CDR).

**Table 1:** Characteristics of subjects with encoding (EF) and retrieval failure (RF) due to mild cognitive impairment

	EF (*n* = 87)	RF (*n* = 78)
Age (years ± std)	73.8 ± 8.3	73.4 ± 6.2
Male/female	39/48	20/58
Education (years ± std)	9.4 ± 5.7	8.0 ± 4.9
MMSE	21.3 ± 6.3	23.4 ± 5.7
CDR	0.5 ± 0.1	0.5 ± 0.1

*CDR* Clinical Dementia Rating, *MMSE* Mini-Mental State Examination

### qEEG sensor-level analysis

**Fig. 2:**
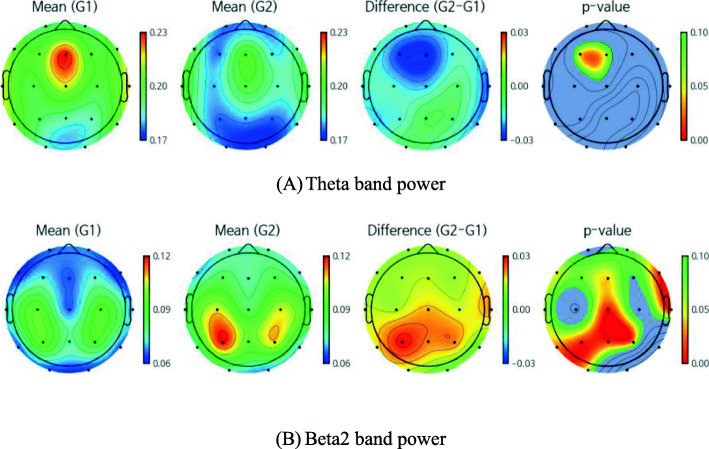
Band power for the encoding failure (G1) and retrieval failure (G2) groups. **a** The EF group showed significantly higher frontal relative theta power than the RF group. A higher average of relative theta power values across all channels was also observed in the EF group. **b** A lower average of relative beta2 power in the frontal, central, temporal, and parietal regions

In the sensor-level analysis, the EF group showed significantly higher frontal theta power than the RF group (Fig. 2a). A higher average of theta power values across all channels was also observed in the EF group, as well as lower beta2 power in the frontal, central, temporal, and parietal regions (Fig. 2b). There were no significant power differences in the delta, alpha1, alpha2, beta1, beta3, and gamma bands between the groups.

### qEEG source-level analysis

**Fig. 3:**
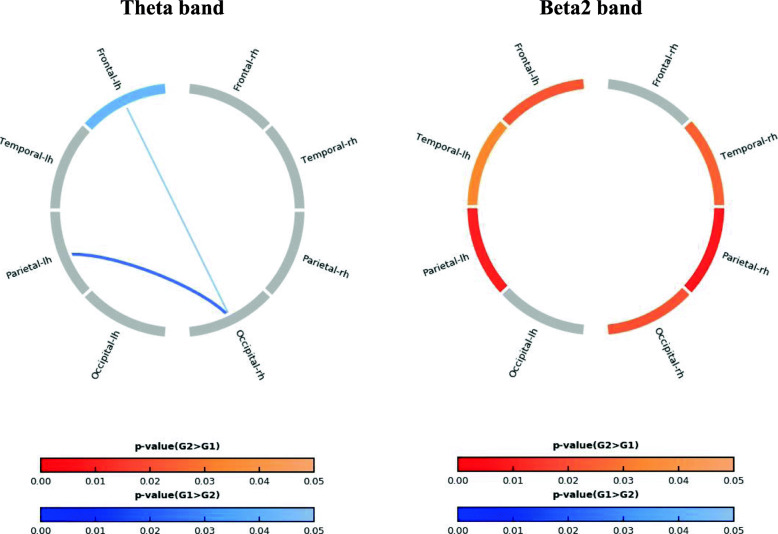
Source ROI power and connectivity between the encoding failure (G1) and retrieval failure (G2) groups. The left panel shows the theta band, and the right panel the beta2 band. The blue color indicates greater significance in the G1 than in the G2 group. Red indicates the opposite

Figure 3 shows the statistically significant difference between the EF and RF groups for the source power of 8 ROIs and the connectivity between the ROIs in the theta and beta2 bands. The theta band power in the left frontal lobe was significantly higher in the EF group. The EF group also showed a higher connectivity in the theta band than the RF group. In contrast, beta2 band power was significantly lower in several ROIs (left frontal, bilateral temporal, bilateral parietal, and right occipital lobe) in the EF group (*p* < 0.05).

### GM volume changes in amnestic MCI

**Fig. 4:**
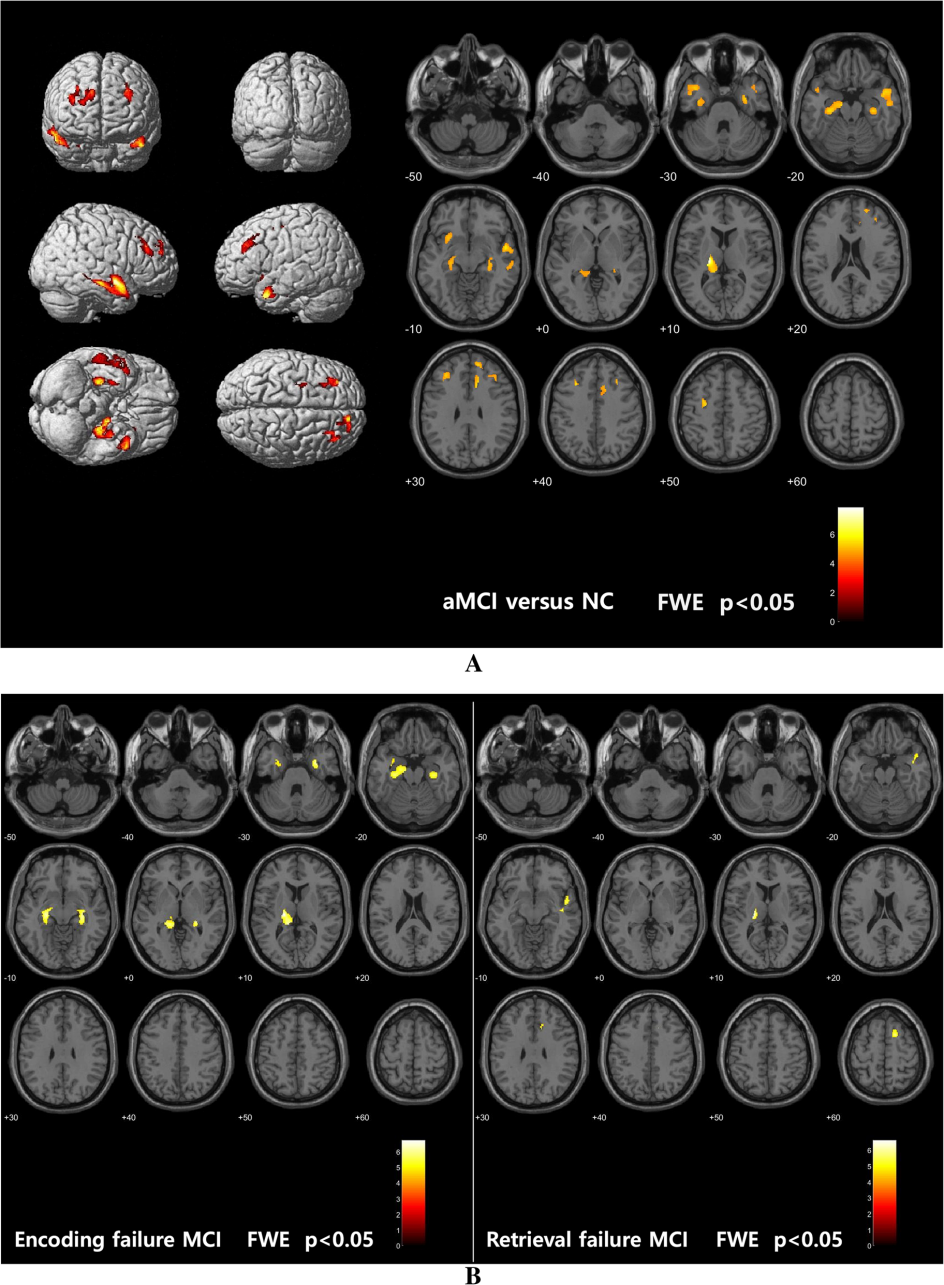
Statistical parametric maps of gray matter volume reductions in aMCI (**a**) and the two subtypes (**b**). FWE, family-wise error; NC, normal control group; aMCI, amnestic mild cognitive impairment

In the VBM analysis, statistically significant atrophic areas in patients with amnestic MCI were overlaid onto an average structural image of the NC group. In amnestic MCI, significant GM atrophy was observed in the left thalamus and precuneus; the bilateral, dorsolateral, and medial temporal areas; the bilateral frontal; and several other areas (Fig. 4a and Table 2). The GM volume reductions underlying EF were located in the left thalamus and the bilateral hippocampi, while those underlying RF were located in the left thalamus, right superior frontal lobe, right superior temporal lobe, and right middle cingulum (Fig. 4b and Table 2). However, the brain volume differences between the RF and EF groups were not significant (uncorrected *p* > 0.005).

**Table 2:** Gray matter volume reductions in amnestic mild cognitive impairment, compared to age-matched normal controls

	*x*, *y*, *z* coordinate			Label	Cluster level		Peak level		
					*p* (FWE-corr)	equivk	*p* (FWE-corr)	*T*	equivZ
Amnestic MCI	21	− 21	12	Thalamus Lt	< 0.001	2324	< 0.001	7.84	7.34
	− 12	− 41	6	Precuneus Lt			< 0.001	5.75	5.54
	− 30	− 21	− 23	Parahippocampus Lt			0.001	5.62	5.42
	53	0	− 11	Temporal Sup Rt	< 0.001	1573	< 0.001	5.70	5.49
	60	− 21	− 14	Temporal Mid Rt			0.001	5.48	5.30
	51	− 9	− 23	Temporal Mid Rt			0.002	5.37	5.19
	12	20	32	Cingulum Mid Rt	< 0.001	567	< 0.001	5.68	5.47
	12	30	33	Cingulum Mid Rt			0.003	5.23	5.07
	14	33	24	Cingulum Mid Rt			0.006	5.09	4.94
	− 29	21	42	Frontal Mid Lt		371	< 0.001	5.66	5.46
	− 29	33	33	Frontal Sup Lt			0.001	5.45	5.26
	− 36	21	33	Frontal Mid Lt			0.009	4.99	4.85
	36	33	24	Frontal Mid Rt		241	0.001	5.63	5.4
	29	27	38	Frontal Mid Rt			0.003	5.23	5.06
	− 45	9	− 24	Temporal Pole Sup Lt		433	0.001	5.53	5.34
	− 27	− 3	48	Precentral Lt		166	0.001	5.47	5.28
	12	51	32	Frontal Sup Medial Rt		328	0.001	5.41	5.23
	18	41	35	Frontal Sup Rt			0.002	5.38	5.20
	23	51	23	Frontal Sup Rt			0.003	5.24	5.07
	33	− 23	− 20	Parahippocampus Rt		621	0.002	5.32	5.15
	30	− 5	− 30	Parahippocampus Rt			0.004	5.19	5.03
	27	− 32	− 3	Hippocampus Rt		225	0.004	5.15	5.00
Encoding failure	− 20	− 20	14	Thalamus Lt	< 0.001	2926	< 0.001	6.7	6.25
	− 12	− 38	6	Hippocampus Lt			< 0.001	6.59	6.15
	− 26	− 23	− 9	Hippocampus Lt			< 0.001	6.34	5.95
	26	− 33	− 3	Hippocampus Rt	< 0.001	879	< 0.001	6.32	5.93
	29	− 26	− 8	Hippocampus Rt			< 0.001	6.12	5.76
	32	− 23	− 17	Hippocampus Rt			< 0.001	5.79	5.49
	30	− 5	− 30	Parahippocampus Rt	0.002	160	< 0.001	6.02	5.68
Retrieval failure	− 21	− 23	9	Thalamus Lt	0.001	201	0	6.61	6.14
	17	11	59	Frontal Sup Rt	0.002	174	0	5.86	5.53
	51	0	− 11	Temporal Sup Rt	< 0.001	390	0.002	5.53	5.24
	44	− 17	− 9	Temporal Sup Rt			0.003	5.41	5.14
	44	− 3	− 18	Temporal Sup Rt			0.018	4.92	4.71
	11	26	36	Cingulum Mid Rt	0.004	111	0.005	5.25	5

FWE, *p* < 0.05. Local maximum more than 8.0 mm apart. *MCI* mild cognitive impairment, *FWE* family-wise error, *Rt* right, *Lt* left, *Sup* superior, *Mid* middle

## Discussion

In this study, we explored functional and structural differences between patients with EF and RF using qEEG and GM volume. The qEEG analysis showed an increase in the theta power spectrum and a decrease in beta2 power in the EF group compared to the RF group. In EEG connectivity analyses, significant differences in iCoh between EF and RF were found in the theta band. Patients with amnestic MCI with EF showed higher theta band connectivity in the frontal-occipital and parietal-occipital connections, compared to those with RF. There was no statistically significant difference in GM volume reductions between the EF and the RF group. However, when compared to the NCs, the VBM analysis demonstrated decreased volumes in the left thalamus and the bilateral hippocampus in the EF but in the right frontal and temporal lobe in the RF group.

The qEEG pattern observed in the EF group in our study was similar to the pattern observed for AD in numerous previous qEEG studies, which showed increased power in low frequency bands (delta and theta) and decreased power in high frequency bands (alpha and beta) [25–31]. A recent study suggested an increase in relative theta power as a first change in patients with AD [25]. During the disease progression of AD, an early increase in theta and decrease in beta is followed by a decrease in alpha and an increase in delta power [32, 33]. Patients with MCI have also shown increases in theta power and decreases in alpha power when compared with normal elderly subjects [28, 33, 34]. Additionally, increased theta power and decreased parietal beta power may predict disease progression to AD in patients with MCI [35, 36]. Another study in non-demented and amyloid-positive subjects showed that higher delta and theta power were associated with clinical progression over time [37]. The patients with amnestic MCI with EF in the present study showed increased theta and decreased beta power when compared with the RF group in our power spectrum and ROI source power analyses. Regarding differences between brain regions at the sensor and source level, we observed an increase in the theta band in the EF group in the frontal area. Previous studies have demonstrated the increased theta power at posterior brain region in predicting AD progression [37, 38]. But, the increased theta in frontal region has also been reported as a predictive factor of clinical progression to AD in several studies [34, 37], and our results are consistent with that. These findings may be associated with an anterior shift in band frequency source [34]. Accordingly, qEEG patterns in patients with amnestic MCI with EF were more similar to the pattern predicting disease progression to AD than in those with RF.

The hallmark of EEG connectivity abnormalities in AD patients is a decrease in coherence of fast rhythms. EEG coherence analyses in patients with AD showed a decrease in connectivity in the alpha frequency band [16, 28, 30, 31, 39, 40]. It has been shown that EEG coherence contributes to the discrimination of AD from normal aging [40] and progression to AD in patients with MCI [38]. Recently, one study reported significant differences in iCoh in the theta and delta bands between groups with progressive and stable MCI, while higher theta coherence was associated with cognitive decline [41]. The significant differences for iCoh were found in the lower frequency bands involving parietal-frontal connections [41]. There is increasing evidence for pathologically increased neuronal activities [42] and connectivity in early AD and MCI [43, 44]. Phase-based measures have also reported increases in the theta band connectivity [43]. Patients with progressive MCI showed higher synchronization than patients with stable MCI [44]. Similar to previous studies, significant differences in iCoh between EF and RF were found in the theta band in our study. Patients with amnestic MCI with EF showed higher theta band connectivity in the frontal-occipital and parietal-occipital connections, compared to those with RF.

In our present study, significant differences in qEEG pattern between EF and RF were found, but there were no significant differences in structural MRI according to cognitive performance. When compared to the NC group, the patients with EF seemed to be more similar to the brain atrophy observed in AD than that observed in patients with RF, but comparing EF and RF separately with controls did not suggest that there are differences between EF and RF. AD is considered a cortical dementia, and structural MRI and qEEG are considered as a marker of neuronal loss and cortical dysfunction [5]. Therefore, we initially expected that the differences between the two groups would be reflected in structural MRI as well as qEEG, and cortical atrophic changes are expected to occur almost simultaneously with EEG abnormalities during AD progression. However, our negative results might suggest that structural MRI may become abnormal a bit later rather than other pathologic biomarkers. Compared with either CSF Aβ1–42 or tau, structural MRI is considered as a bit later biomarker [45, 46], and sometimes, abnormal EEG pattern may be observed earlier than structural MRI [47]. It may be difficult to compare directly what is the earlier biomarker between qEEG and structural MRI, but further research is needed regarding this. And, it will be needed to investigate if there is a difference between the two groups through a pathologic biomarker, an earlier biomarker (either CSF Aβ1–42 or tau).

## Limitations

The current study was subject to several limitations. Since this study is a cross-sectional study, it cannot be confirmed that patients with amnesiac MCI with EF actually progress to AD. It is just assumed that amnestic MCI patient with EF is more likely to progress to AD than patient with RF, as the patterns of qEEG, EEG connectivity, and cortical atrophy in the EF group were more similar to the patterns observed for AD in numerous previous studies. Therefore, longitudinal follow-up studies may be needed to investigate whether patients with amnestic MCI with EF can progress to AD. Second, gender is not considered as covariate in analyses, although there was significant difference between the EF and RF groups in gender ratio (*p* = 0.010). Lastly, it may be difficult to accurately estimate ROIs by calculating using 21 channels. However, EEG in our study was generally performed in clinical practice rather than for research. Similar methods were used in some previous studies using clinical data [48].

## Conclusions

Our findings indicate that patients with amnestic MCI with EF and those with RF differ functionally, and that both show different brain atrophy sites in comparison to NCs. By integrating power spectral, EEG coherence, and MRI volumetric analyses, we found that patients with EF due to amnestic MCI show a pattern that is more consistent with the prodromal stage of AD than the pattern observed in patients with RF. Prospective follow-up studies are needed to investigate whether different types of memory impairments can predict the underlying pathology of amnestic MCI.

## Supplementary Information

**Additional file 1.** Band power for the encoding failure (G1) and retrieval failure (G2) group.

**Additional file 2.** Source ROI power and connectivity between the encoding failure (G1) and retrieval failure (G2) groups.

## Data Availability

The datasets generated and/or analyzed during the current study are not publicly available due to data protection regulations, but are accessible at the corresponding author on reasonable request.

## Abbreviations

MCI: Mild cognitive impairment
AD: Alzheimer’s disease
EF: Encoding failure
RF: Retrieval failure
qEEG: Quantitative electroencephalography
MRI: Magnetic resonance imaging
MMSE: Mini-Mental State Examination
K-IADL: Korean version of the instrumental activities of daily living scale
iCoh: Imaginary part of coherency
GM: Gray matter
VBM: Voxel-based morphometry
amICA: Adaptive mixture independent component analysis
WM: White matter
CSF: Cerebrospinal fluid
TIV: Total intracranial volume
DARTEL: Diffeomorphic Anatomic Registration Through Exponentiated Lie
CAT12: Computational Anatomy Toolbox
SPM12: Statistical Parametric Mapping software
