# Supplementary figures and images for: Differences between memory encoding and retrieval failure in mild cognitive impairment: results from quantitative electroencephalography and magnetic resonance volumetry
Source: Alzheimers Res Ther. 2021 Jan 4;13:3. doi: 10.1186/s13195-020-00739-7 (PMC7784298; doi:10.1186/s13195-020-00739-7)

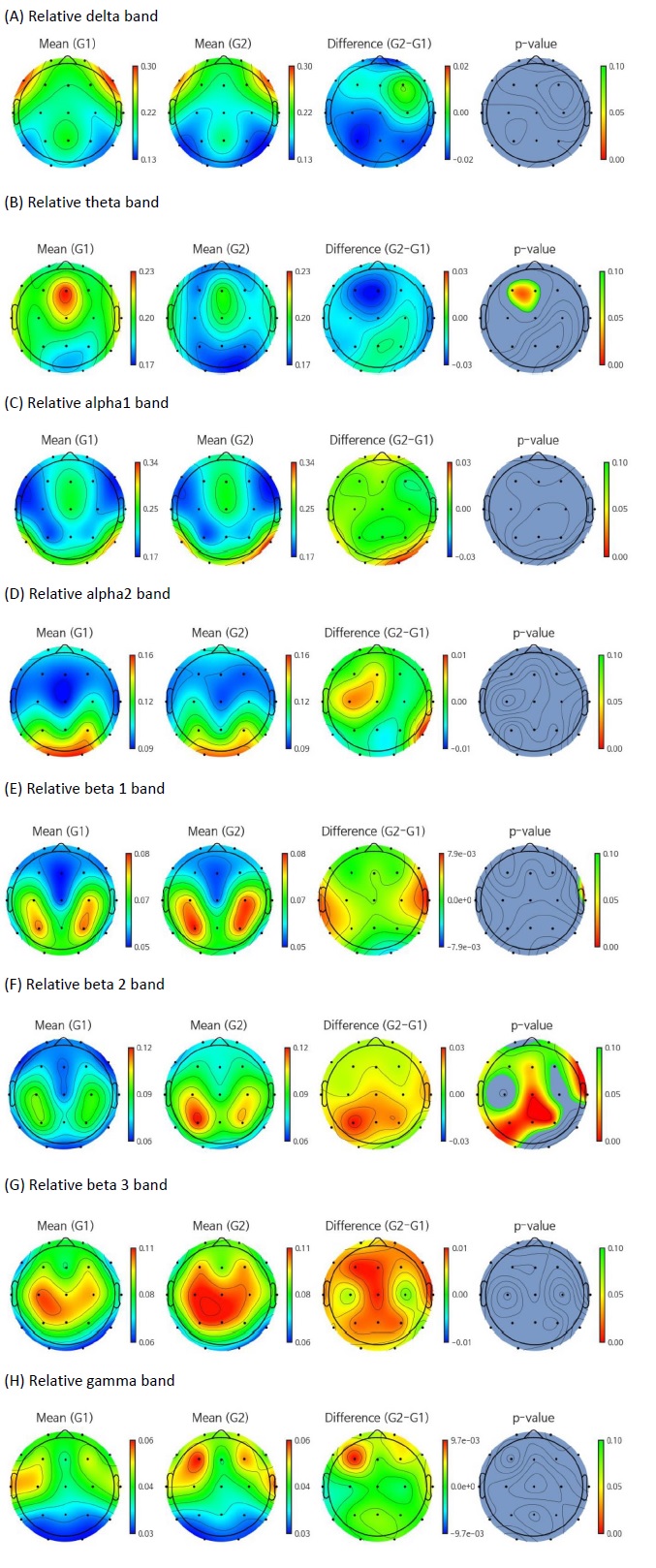

Supplement: Supplementary file 1 — Additional file 1. Band power for the encoding failure (G1) and retrieval failure (G2) group. [file 13195_2020_739_MOESM1_ESM.jpg]

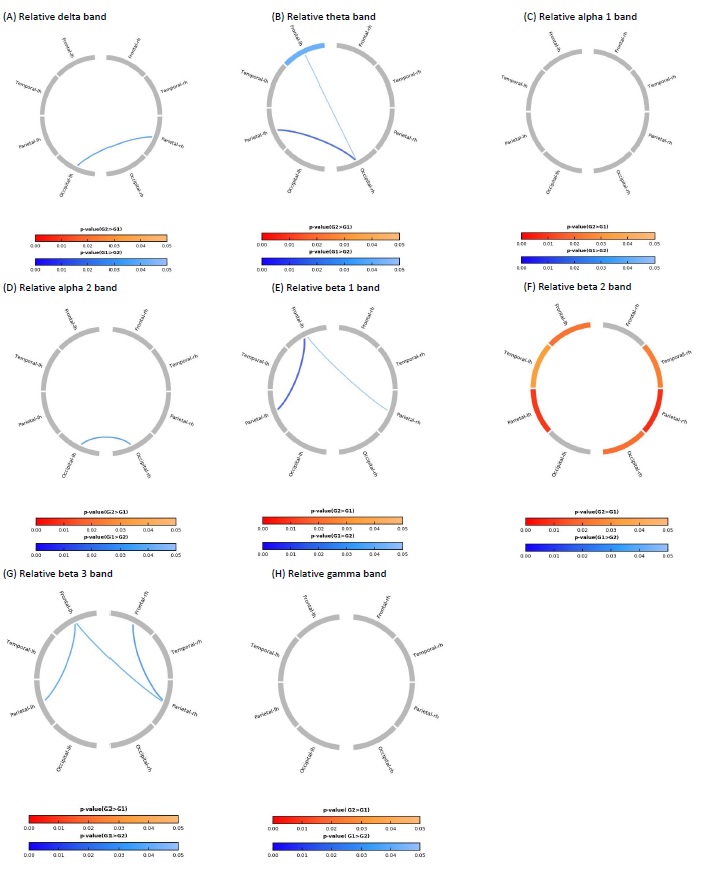

Supplement: Supplementary file 2 — Additional file 2. Source ROI power and connectivity between the encoding failure (G1) and retrieval failure (G2) groups. [file 13195_2020_739_MOESM2_ESM.jpg]
